# Effect of cytotoxic CD8+ T-cells secretory proteins on hypoxic pancreatic cancer cells

**DOI:** 10.1371/journal.pone.0311615

**Published:** 2025-01-30

**Authors:** Eiman Abdo, Mohammad A. Ismail, Sabal Al Hadidi, Mairvat Al-Mrahleh, Tareq Saleh, Malik Zihlif, Nidaa A. Ababneh

**Affiliations:** 1 Department of Pharmacology, School of Medicine, University of Jordan, Amman, Jordan; 2 Cell Therapy Center, The University of Jordan, Amman, Jordan; 3 Faculty of Medicine, Department of Pharmacology and Public Health, The Hashemite University, Zarqa, Jordan; The University of Texas MD Anderson Cancer Center, UNITED STATES OF AMERICA

## Abstract

**Background:**

Hypoxia in tumor cells is linked to increased drug resistance and more aggressive behavior. In pancreatic cancer, the tumor microenvironment is notably hypoxic and exhibits strong immunosuppressive properties. Given that immunotherapy is now approved for pancreatic cancer treatment, further understanding of how pancreatic tumor cell hypoxia influences T-cell cytotoxicityis essential.

**Objective:**

This study examined how hypoxia affects the interaction between pancreatic tumor cells (PANC-1) and cytotoxic CD8+ T-cells.

**Methods:**

Pancreatic tumor cells (PANC-1) were exposed to 20 cycles of chronic hypoxic conditions, each for 72 hours, followed by a re-oxygenation period for 24 hours. On cycles 10 and 20, PANC-1 conditioned media (CM) was harvested, and the hypoxic PANC-1 cells were co-cultured with either the activated cytotoxic CD8+ T-cells or with CD8+ T-cells CM. CD8+ T-cells CM was collected after five days of cell activation using anti-CD3/CD28 antibodies and interleukin-2 (IL-2). CD8+ T-cells were activated for 72 hours and then cultured with the hypoxic PANC-1 CM.

**Results:**

Hypoxic PANC-1 cells showed significant resistance to the lytic effect of either CD8+ T-cells co-culture or CD8+ T-cells CM treatment compared to normoxic PANC-1 cells. A significant decrease in TNF-α and IFN-γ levels was also detected. Additionally, a significant increase in IL-6, p53 and TNF-α gene expression levels was observed in PANC-1 cells treated with CD8+ T-cells CM. Moreover, IL-6 gene expression level showed a significant difference between hypoxic and normoxic PANC-1 cells. CD8+ T-cell proliferation and cytokines production were significantly higher in cells co-cultured with PANC-1 CM. However, no significant differences were observed after treatment with either hypoxic or normoxic PANC-1 CM.

**Conclusion:**

Hypoxia decreases PANC-1 cells’ sensitivity to cytotoxic CD8+ T-cells. Reduced tumor cell susceptibility to CD8+ T-cells was associated with increased IL-6 expression and reduced TNF-α and IFN-γ levels. Thus, cytokine dysregulation might contribute to the hypoxia-mediated resistance of pancreatic tumor cells to CD8+ T-cells.

## Introduction

Pancreatic cancer (PC) is characterized by a high mortality rate, poor prognosis and a high rate of relapse [1]. The PC five-year survival rate is approximately 30% in early-stage patients, decreasing to only 3% in patients with metastatic disease. Pancreatic ductal adenocarcinoma (PDAC) accounts for approximately 75%-95% of all PC types [2]. Adjuvant therapy of gemcitabine or gemcitabine-combined therapy is the first-line pharmacological treatment of PDAC and has previously shown variable outcomes on patients’ survival [3,4]. This is partly because PDAC is associated with higher rates of chemoresistance [5]. Several factors contribute to the development of chemoresistance in PDAC, including pharmacological resistance, stemness, unfavorable tumor microenvironment (TME) conditions, and higher rates of tumoral hypoxia [6–8].

The development of tumoral hypoxia, or oxygen deprivation, contributes significantly to acquiring more malignant traits [9]. PC is associated with remarkable levels of hypoxia in comparison to other solid tumors [10]. Pancreatic tumoral hypoxia results in adaptive alterations in both malignant cells and their surrounding stroma, leading to cancer progression and therapy resistance [11–13]. Moreover, tumoral hypoxia is associated with extremely immunosuppressive features [14]. Subsequently, immunotherapy has not shown significant clinical success despite its recent approval for the treatment of PDAC [15]. Neither monotherapy of programmed cell death ligand1 (PD-L1) blockers or cytotoxic T-lymphocyte-associated antigen 4 (CTLA4) inhibitors nor combination therapy were impactful in PDAC treatment [13]. Unfortunately, this could be partly because the majority of preclinical studies were conducted in the absence of hypoxic conditions [16].

To explore the impact of hypoxic conditions in the tumor microenvironment on tumor-immune cell interactions, this study utilized a previously validated model of tumor cell hypoxia to investigate the crosstalk between hypoxic pancreatic cancer cells (PANC-1) and cytotoxic CD8+ T-cells [17–19] Additionally, this study aimed to identify the cytokines involved in modulating the effects of cytotoxic CD8+ T-cells under hypoxic conditions, which may contribute to immunotherapy resistance in pancreatic cancer. While several previous studies have explored tumor-host interactions and the immunosuppressive mechanisms within the tumor microenvironment, to our knowledge, no in vitro studies have specifically investigated the crosstalk between hypoxic PANC-1 cells and CD8+ T-cells.

## Methodology

### Cell culture

The human pancreatic cancer cell line (PANC1) was purchased from the ATCC (American Type Culture Collection, CRL-1469). PANC1 cells were cultured in Advanced Dulbecco’s Modified Eagle’s Medium (ADMEM) supplemented with 10% fetal bovine serum (FBS), 1% Antibiotic-Antimycotic and 1% Glutamax (all from Gibco) at 37°C and 5% CO_2_. PANC1 cells were subcultured upon reaching 80% confluency and detached using TrypLE (1X). Pancreatic cancer cells (PANC1) were exposed to 10 and 20 shots of cycling hypoxia using anaerobic sachets (AnaeroGen Compact, Oxoid), and hypoxia was validated as described previously [17–19]. Each cycling shot comprised 72 hours under hypoxic conditions and 24 hours of re-oxygenation at 37°C and 5% CO_2_. The sachets consist of ascorbic acid and activated carbon to absorb the O_2_ rapidly upon exposure in the pouch, and within 30 minutes, the O_2_ concentration can reach below 1%. Cells under normoxic conditions were kept in the same incubator at 5% CO_2_, 21% O_2_, and 37°C to control the experiment.

### PBMCs isolation

Fresh human peripheral blood samples were collected in EDTA tubes from three healthy unrelated donors, following Cell Therapy Center (CTC) Institutional Review Board (IRB) committee approval (7/11/2019, number (IRB/7/2019). The donors signed written informed consent for their participation in the study. Peripheral blood mononuclear cells (PBMCs) were isolated by Ficoll-Paque Premium solution with 1.077 density according to the manufacturer protocol (Sigma). In brief, blood samples were mixed with an equal volume of PBS, and then 10 ml of diluted blood samples were mixed gently with five ml Ficoll-Paque solution without mixing to separate layers between Ficoll-Paque and blood cells. Density gradient centrifugation was carried out at 450 g for 30 minutes at room temperature (RT) and break 0 to produce four layers representing different cell types. The mononuclear cell layer was drawn and cultured in RPMI-1640 medium containing 10% FBS, 1% Antibiotic-Antimycotic and 1% Glutamax. Cells were maintained overnight in the incubator at 37°C and 5% CO_2_.

### CD8+ T-cells isolation

Peripheral blood CD8+ T-cells were isolated by magnetic sorting using a CD8 Microbeads kit from Miltenyi Biotech [20]. The cell isolation buffer was prepared based on the manufacturer’s protocol by mixing 1X PBS, 0.5% FBS and 2 mM EDTA. PBMCs were incubated with CD8 microbeads for 15 minutes at 2–8°C. Then, cells were centrifugated, resuspended with the isolation buffer, and applied on a column in a magnet separator. The separated CD8 cells on microbeads were harvested and maintained in RPMI-1640 medium containing 10% FBS, 1% Antibiotic-Antimycotic, 1% Glutamax and kept at 37°C and 5% CO_2_.

### Flow cytometric characterization of CD8+ T-cells

Flow cytometry analysis assessed the purity of the isolated CD8+ T-cells using antibodies against the following cell surface markers: CD3, CD8, and CD4. Cells were centrifuged at 300 g for 5 minutes and resuspended in 600 μl PBS. After that, cells were split into three tubes, stained with 10 μl of diluted primary antibodies (1/20 μl in PBS), and then incubated for 10 minutes in the dark at RT. Then, samples were analyzed using BD FACSCanto II flow cytometry to measure the percentage of the isolated CD8+ T-cells.

### CD8+ T-cells counting after isolation

The isolated CD8+ T-cells were counted based on their percentage in PBMCs and the eluted unwanted cells after isolation. To detect the percentage of CD8+ T-cells, samples from the isolated PBMCs and unwanted cells were stained with 10 μl of diluted CD8 antibodies and read on flow cytometry. PBMCs and unwanted cell counting were performed by staining the cells with trypan blue and counted using the Countess II instrument.


CD8+T-cellscount=(PBMCcount*percentageofCD8+T-cells)–(unwantedcellscount*percentageofCD8+T-cells).

CD8+ T-cells were counted after isolation using flow cytometry analysis (S1 Fig). The mean percentage of CD8+ T-cells in PBMCs was 40%, and the mean rate of eluted CD8+ T-cells in unwanted cells during the isolation was 10%. PBMCs and eluted unwanted cells were counted using Trypan blue. We used the following formula to calculate the mean number of CD8+ T-cells in 50 ml of whole blood:

CD8+T-cellcount=(PBMCscount*percentageofCD8+T-cells)—(elutedunwantedcellscount*percentageofCD8+T-cells).

### CD8+ T-cells viability

Cell viability was measured using 7-Aminoactinomycin D (7-AAD) dye that penetrates the non-viable cells and binds to DNA, producing a fluorescent signal that is detected via flow cytometry. Approximately 200 μl of cultured CD8+ T-cells were centrifuged and resuspended in 200 μl PBS, then stained with 5 μl 7-AAD and incubated for 10 minutes in the dark at RT. Then, samples were analysed using BD FACSCanto II flow cytometry.

### CD8+ T-cell activation/expansion

The ImmunoCult^™^ Human CD3/CD28 T-Cell Activator kit with rhIL-2 was used for CD8+ T-cells activation and expansion. Briefly, 100 IU of rhIL-2 were mixed with 25 μl of CD3/CD28 antibodies solution and mixed with 1x10^6^ CD8+ T-cells culture.

### Collection of conditioned media (CM)

Conditioned media (CM) were collected over hypoxic PANC1 cells and CD8+ cell cultures. PANC-1 hypoxic CM (HCM) was collected directly after 72 hours of the hypoxic shot on cycles 10 and 20. Also, the normoxic CM (NCM) was collected from normoxic cell culture to serve as a control. Then, CMs were filtered using a sterile 0.22 μm filter to remove cell debris. Aliquots of the CM were stored at -80°C for further use. Regarding CD8+ T-cells CM, cells were activated for five days with CD3/CD28 antibodies and rhIL-2. After that, the CM was collected by centrifugation at 300 g for 5 minutes and then filtered using a sterile 0.22 μm filter. Aliquots of the CM were stored at -80°C for further use.

### CM treatment of PANC1 or CD8+ cells

After three days of CD8+ T-cell activation, cells were cultured in either HCM or NCM derived from PANC-1 cell culture in 50% and 100% ratios, respectively. Cells were seeded in a 24-well plate at a density of 500,000 cells/well and incubated with HCM and NCM for 72 hours. Afterwards, cell culture media (CCM) was collected for cytokine analysis. Activated CD8+ T-cell culture media was collected to serve as a negative control. On the contrary, hypoxic and normoxic PANC-1 cells at cycles 10 and 20 were cultured in CD8+ T-cells CM collected after five days of activation. PANC-1 cells were seeded in 6-well plates at 300,000 cells/well density. After 24 hours, CCM was aspirated and replaced with different CD8+ T-cells CM ratios: 0, 25%, 50%, 75%, and 100%. After 72 hours of treatment, the supernatant was collected for cytokines analysis, and PANC-1 cells were harvested for qRT-PCR assay. Untreated PANC-1 cells were cultured in RPMI-1640 media, and the CM was used as a control.

### Co-culture assay

Co-cultures of either hypoxic or normoxic PANC-1 cells (target cells) and activated CD8+ T-cells (effector cells) were performed after activating CD8+ T-cells for 72 hours. PANC-1 cells were seeded in 6-well plates at 300,000 cells/well density for 24 hours. After that, media was aspirated, and activated CD8+ T-cells were suspended in 2 ml of RPMI-1640 media/well and added to the attached hypoxic and normoxic PANC1 cells at the following ratios: 20:1, 10:1, and 5:1, and incubated for 72 hours at 37°C and 5% CO_2_. Controls of either PANC1 cells only or activated CD8+ T-cells were cultured in RPMI 1640 media and used as normal controls. Then, the CM of the co-culture samples and the control cells were collected for cytokine analysis and centrifuged to remove cell debris. The attached PANC1 cells were harvested for qRT-PCR analysis.

### T-cell proliferation and counting after activation

5-(and 6)-Carboxyfluorescein diacetate succinimidyl ester (CFSE) staining was used to measure CD8+ T-cell activation and proliferation. CFSE is cleaved intracellularly by an esterase enzyme to a fluorescent dye, which binds covalently to intracellular molecules. Upon cell division, the dye can be uniformly diluted between the generated cells, detecting 8–10 generations. Briefly, CD8+ T-cells were centrifuged and re-suspended in one ml of 5 μM CFSE in the dark and incubated for 20 minutes at 37°C and 5% CO_2_. Then, CFSE dye was quenched by adding 5 ml serum-containing media, and cells were centrifugated and resuspended in RPMI 1640 media and maintained in the incubator at 37°C with 5% CO_2_. For the co-culture assay and after CD8+ T-cells activation for three days, CFSE dye fluorescence was measured to calculate the mean of CD8+ T-cells number of divisions and folds increase based on the following formula:

$$MFI(stimulatedcells)=MFI\left(unstimulated\;cells\right)/2^{PS}$$

Then, cell counting was performed by multiplying the original number of cells * fold changes.

Where MFI indicates the mean fluorescent intensity for CFSE, PS (proliferation score) is the mean number of divisions of stimulated CD8+ T-cells relative to the mean number of unstimulated cells [21]. For HCM and NCM cultures and after three days of CD8+ T-cell activation, the cells were cultured in HCM and NCM of PANC1 cells in 50% and 100% ratios for 72 hours. Then, CFSE dye fluorescence was read to measure the CD8+ T-cell proliferation.

### Viability assay by MTT

MTT (3–4,5-dimethyl-thiazol-2-yl 2,5-diphenyl-tetrazolium bromide) (MTT) assay was carried out to study the impact of CD8+ T-cells on hypoxic PANC1 cells after exposing them to 10 and 20 shots of hypoxia. Briefly, cells were seeded in a 96-well plate at 7000 cells/well density. After 24 hours, the culture media was aspirated, and the hypoxic and normoxic cells were treated with either CD8+ T-cell CM, or co-cultured with activated CD8+ T-cells. Untreated cells cultured only with advanced DMEM media, or RPMI-1640 media were used as negative controls. For the co-culture experiment, 10 μl MTT was added and incubated for 4 hours, then media was aspirated, and 50 μl of solubilizing agent was added to dissolve the formazan and measure the optical density at 570 nm. The absorbance was directly proportional to the number of viable cells. CD8+ T-cells CM treatment was performed using increasing ratios of CM 25%, 50%, 75%, and 100% for hypoxic and normoxic PANC1 cells. In the co-culture assay, different ratios of the effector: target cells were used (20:1, 10:1, 5:1). The cytotoxicity of CD8+ T-cells and their CM was calculated in terms of the percent of viable cells in comparison to the untreated cells.

### RNA extraction and quantitative real-time polymerase chain reaction (qRT-PCR)

Total RNA was extracted from the hypoxic and normoxic PANC1 cells treated with CD8+T-cells CM using RNeasy Minikit (QIAGEN) according to the manufacturer’s protocol. The concentration and purity of the extracted RNA were checked on a nanodrop spectrophotometer. Then complementary DNA (cDNA) was prepared by reverse transcription using QuantiTect Reverse Transcription Kit (QIAGEN). A reverse transcription reaction was performed according to the manufacturer’s instructions, and cDNA samples were stored at -20 °C for further use. Quantitative RT-PCR was performed using QuantiTect SYBR Green PCR kit (QIAGEN), and samples were run on CFX thermocycler (Bio-Rad). The reaction was performed by initial denaturation at 94°C for 15 minutes. Then, 40 cycles of denaturation at 95°C for 15 seconds, annealing for 30 seconds at a specific temperature for each primer set (S1 Table), and extension at 72°C for 30 seconds. GAPDH was used as a normalizing gene.

### LEGENDplex Multi-Analyte cytokine array

Cytokine array was carried out on treated CD8+ T-cells and treated PANC-1 using LEGENDplex Multi-Analyte Flow Assay Kit. Samples were prepared according to the manufacturer’s instructions. In brief, after CM centrifugation to remove cell debris, capture beads were added and incubated on a plate shaker for two hours at RT. After that, centrifugation and washing steps were applied to remove any background noise. Next, the detection antibodies cocktail was added and incubated in the dark at RT for one hour. Finally, Streptavidin-phycoerythrin (SA-PE) was added, bound to the detection antibodies, and incubated for 30 minutes on a plate shaker at RT. Measured fluorescent intensity represents the number of targeted analytes in the sample. To determine the concentration of each cytokine, a standard curve of seven diluted standards supported by the kit was prepared, ranging between 2.44 pg/ml to 10,000 pg/ml, with a sample of zero concentration. Samples were transferred to 5 ml FACS tubes and read on BD FACSCanto II flow cytometry. Three standard curves were obtained for the three detected cytokines TNF-α, IFN-γ and IL-6 with LOD values 0.48, 0.33, and 6.49, respectively. R2 values and standard curves are plotted in Fig 5. Data analysis was generated using Biolegend’s LEGENDplex data analysis software, which the manufacturer supported. The x-axis represents analyte concentrations (pg/ml), and the y-axis represents the mean fluorescent intensity for each analyte.


Thepercentageofconcentrationchange(%)=theconcentrationofthetreatedsample/theconcentrationoftheuntreated(control)sample*100%.

### Data analysis

Statistical analysis was carried out using GraphPad Prism-8 software. All experiments were done in triplicates. Statistical differences between different treatments and differences in cytokine production were measured based on multiple t-test analyses, and a significant difference was considered at a P-value <0.05.

## Results

### CD8+ T-cells purity assessment

The main experimental approach of this work was to establish a co-culture of pancreatic tumor cells and CD8+ T-cells under hypoxic conditions. First, CD8+ T-cell isolation and activation were performed as described in the Methods. CD8+ T-cells were counted after activation based on CFSE analysis and measuring the mean number of divisions. For the co-culture experiment, CFSE was read after three days of activation. The mean number of divisions was 0.25, and the fold increase in cell number was 1.20. T-cell purity was checked by flow cytometry after cell isolation from the donors (S1 Fig). The mean percentage of the isolated CD8+ T-cells was 99.0%, and the CD3+ T-cells were also 99.0%, while the mean percentage of CD4+ T-cells was less than 0.5%. Cell purity was also assessed after three days of CD8+ T-cell activation. The purity of CD8+ T-cells was 99.0% and 0.5% for CD4+ T-cells (S1 Fig). On day 5 of CD8+ T-cells atyhjh ctivation, the purity was also 99.0% (S1 Fig).

### Effect of PANC1-derived CMs on the proliferation and viability of activated CD8+ T-cells

**Fig 1:**
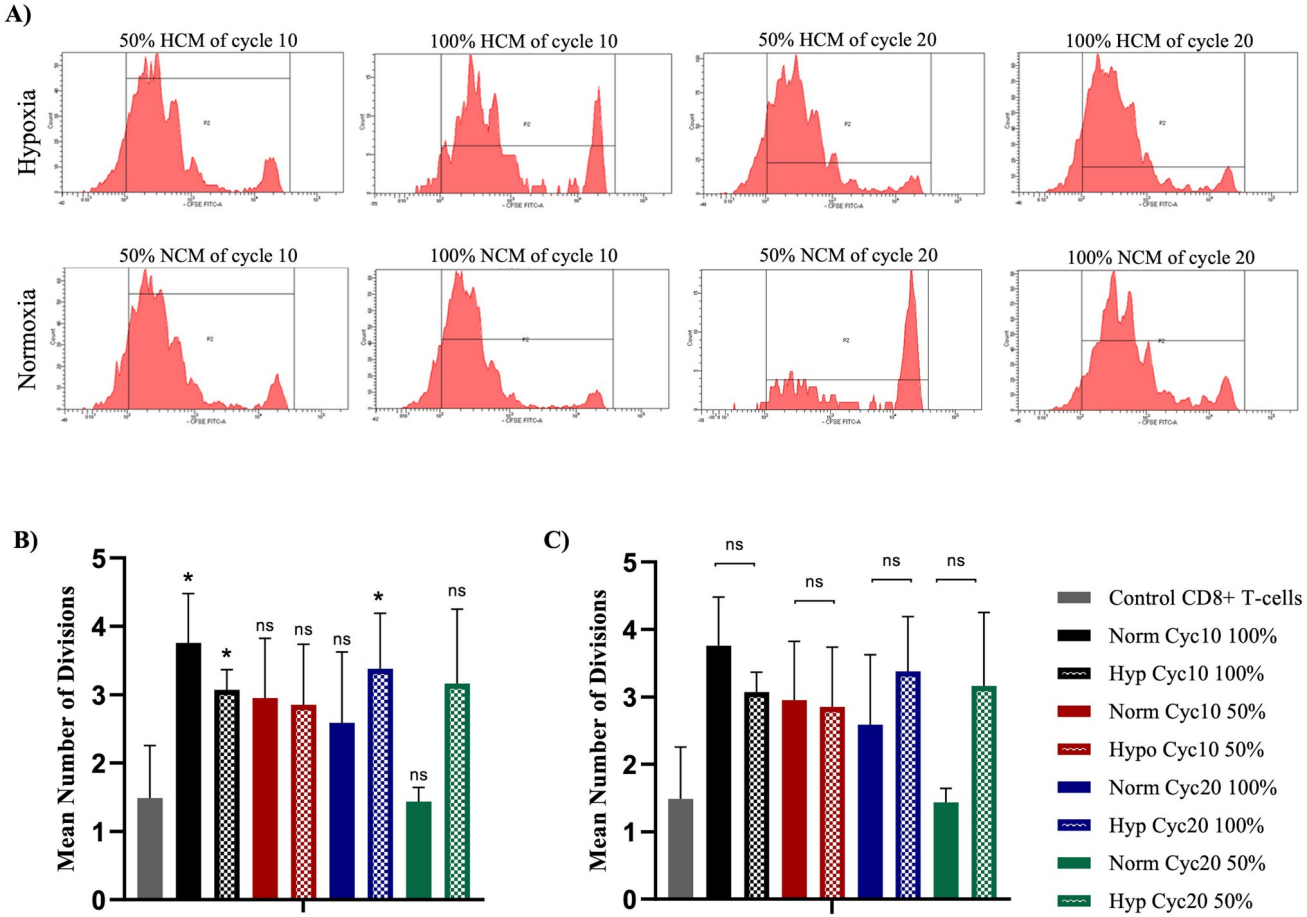
CFSE staining of CD8+ T-cells for proliferation assessment. **A)** Histograms of CFSE-stained CD8+ T-cells treated with different percentages of HCM or NCM after 10 and 20 cycles of hypoxia. **B)** The mean number of divisions for CD8+ T-cells cultured on HCM or NCM compared to untreated CD8+ T-cells. **B.** Comparison of the mean number of divisions between HCM and NCM-treated CD8+ T-cells (*P<0.05).

To determine if hypoxic conditions can facilitate an immunosuppressive effect of PANC-1 cells against cytotoxic CD8+ T-cell proliferation, CD8+ T-cells were activated for three days. Then, they were cultured using 50% and 100% ratios of HCM or NCM. HCM was derived from PANC-1 cells exposed to 10 and 20 cycles of hypoxia for 72 hours, while NCM was derived from PANC-1 cells cultured under normoxic conditions. Validation of hypoxia markers under these conditions was demonstrated previously [17–19]. A control CD8+ T-cell culture media was used as a negative control. CFSE dye was used to measure the mean number of CD8+ T-cell divisions, significantly higher in cells treated with 100% hypoxic and normoxic CM than in untreated cells. However, no significant difference between cells treated with HCM or NCM was observed in all ratios (Fig 1A and 1B). After treating CD8+ T-cells with HCM and NCM, cell viability was assessed using the 7-AAD assay. The percent of cells stained with 7-AAD represents the non-viable cells. All CD8+ T-cell viability measurements were more than 95% when cultured in RPMI-1640 media, NCM or HCM. The mean percentages of non-viable cells are shown in S2 Fig.

### Effect of CD8+ T-cells co-culture and CM on hypoxic and normoxic PANC-1 cell viability

**Fig 2:**
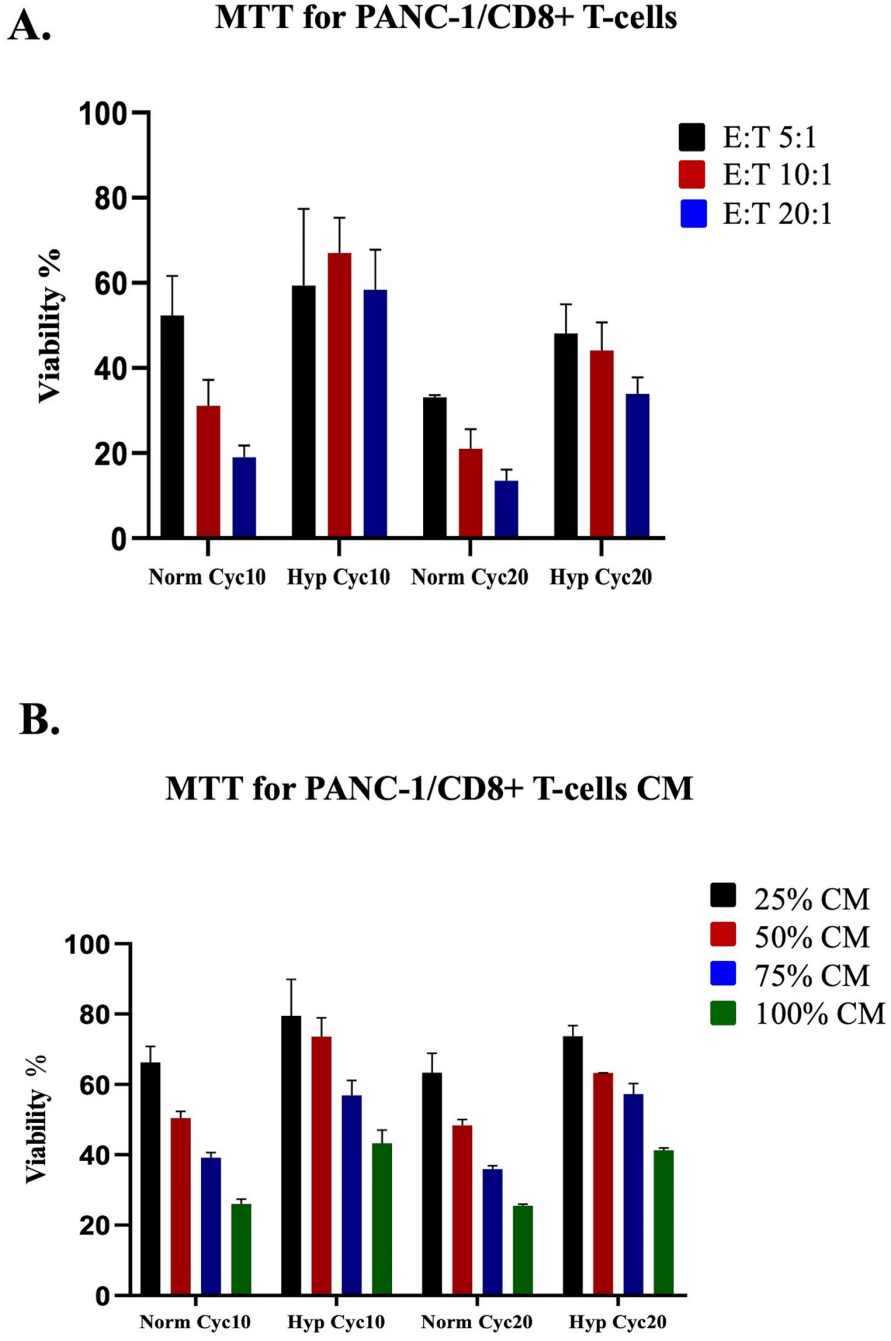
Effect of CD8+ T-cells coculture and CM on hypoxic and normoxic PANC-1 cells. **A.** Mean percentage of cell viability in hypoxic and normoxic PANC-1 cells co-cultured with CD8+ T-cells for 72 hours. **B.** Percentage of cell viability in hypoxic and normoxic PANC-1 cells cultured with CD8+ CM for 72 hours. Percent viability was normalized relative to control (normoxic) PANC-1 cell viability.

A co-culture experiment was performed to test the sensitivity of hypoxic PANC-1 cells to cytotoxic CD8+ T-cells, and an MTT assay was carried out to measure cell viability. CD8+ T-cells were collected from the donors and activated for three days, then co-cultured for 72 hours with either hypoxic PANC-1 cells, previously exposed to 10 or 20 hypoxic shots, or with normoxic cells in an effector: target ratio (E:T; 20:1, 10:1 or 5:1). Non-co-cultured hypoxic and normoxic PANC-1 cells were used as controls. Interestingly, a significantly higher viability rate was recorded in hypoxic PANC-1 cells relative to their normoxic counterparts at ratios E:T 20/1 and 10/1 after ten cycles of hypoxia, suggesting that the CD8+ T-cells cytotoxic effect positively correlates with E:T ratios (Fig 1A). Moreover, hypoxic cells generated after 20 cycles of chronic hypoxia showed a statistically significant increase in viability at all ratios (Fig 2A). Then, isolated CD8+ T-cells were activated with CD3/CD28 antibodies and rhIL-2 for five days, and then the CM was collected. MTT assay measured cell viability after culturing the hypoxic PANC-1 cells with different ratios of CD8+ T-cells CM (100%, 75%, 50%, and 25%). Untreated normoxic and hypoxic cells were used as controls. The resulting lytic effect on PANC1 cells was directly correlated with an increasing concentration of CM. Additionally, hypoxic cells displayed statistically significant higher resistance to activated CD8+ T-cells secretory proteins in cells that underwent 10 cycles of hypoxia and treated with 100%, 75%, and 50% of CM, as well as cells that underwent 20 cycles of hypoxia at all ratios (Fig 2B). These results suggest that tumoral cell hypoxia can interfere with the cytotoxic ability of activated T-cells.

### Gene expression alterations in hypoxic and normoxic PANC-1 cells co-cultured with CD8+ T-cells

**Fig 3:**
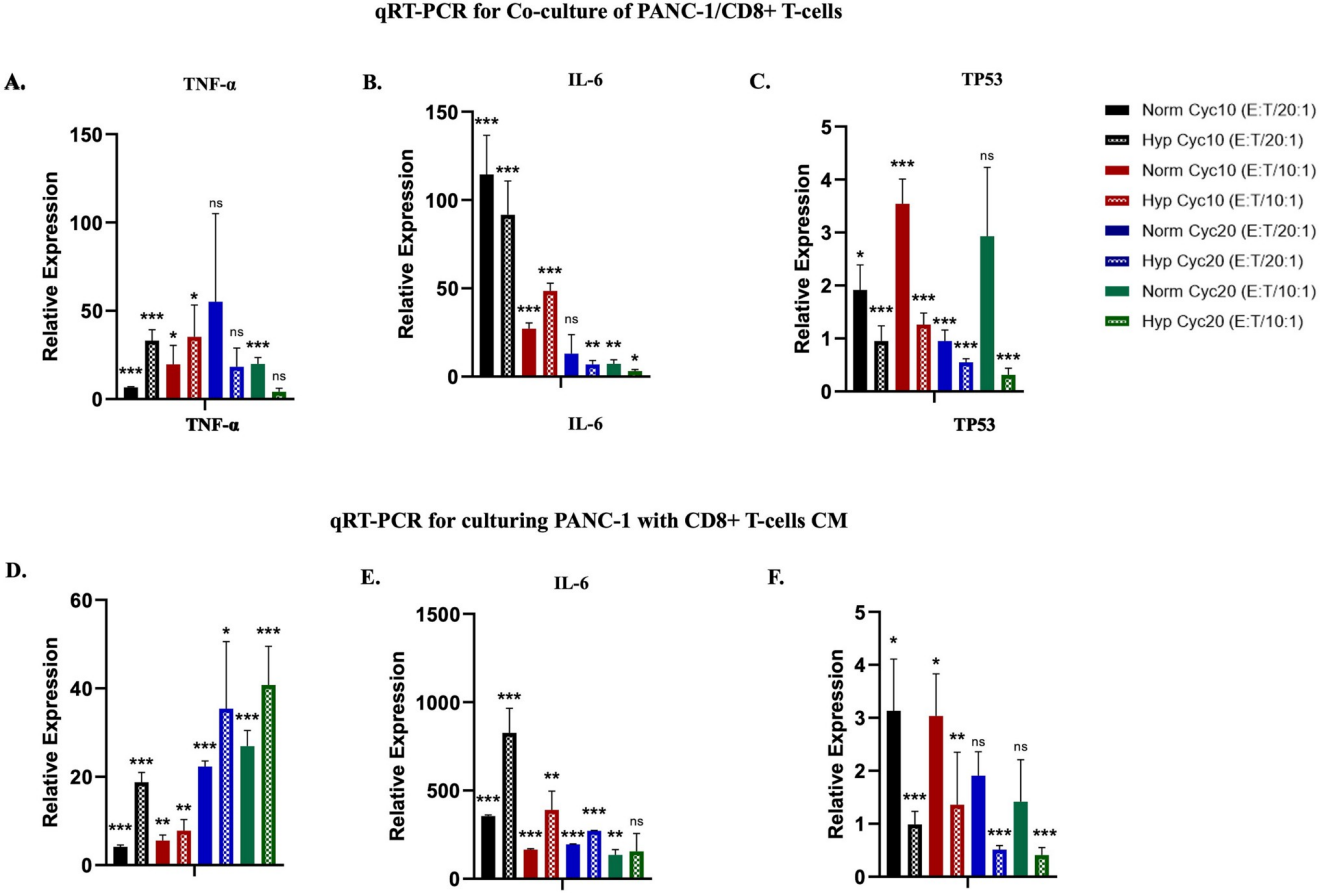
Relative expression levels in TNF-α, IL-6, and p53 in co-culture and CM treatment of hypoxic and normoxic PANC-1 cells with CD8+ T-cells. **A.** TNF-α P-value for treated PANC-1 cells vs. control. **B.** IL-6 P-value for treated PANC-1 cells vs. control. **C.** TP53 P-value for treated PANC-1 cells vs. control. (*P<0.05, **P<0.01, ***P<0.001). **D.** TNF-α P-value for treated PANC-1 cells vs. control. **E.** IL-6 P-value for treated PANC-1 cells vs. control. **F.** TP53 P-value for treated PANC-1 cells vs. control. (*P<0.05, **P<0.01, ***P<0.001).

qRT-PCR was used to assess gene expression of p53, TNF-α, and IL-6 in hypoxic and normoxic PANC-1 cells after cycle 10 and 20 of hypoxia (Norm cyc10, Hyp cyc10, Norm cyc20, and Hyp cyc20). p53 is known for its established role in regulating hypoxia and, importantly, the resultant resistance to chemotherapy, especially in pancreatic tumor cells [22,23]. Both TNF-α and IL-6 are critical inflammatory mediators implicated in regulating the immune response against hypoxic cells [24]. PANC-1 cells were first co-cultured with CD8+ T-cells in an E:T ratio of 20:1 and 10:1. A statistically significant increase in *TNFα* expression was observed in all hypoxic and normoxic PANC-1 cells after 10 cycles of hypoxia, and only under E:T ratio of 10:1 after 20 cycles of hypoxia. Similarly, a significant difference was observed between treated hypoxic and normoxic cells at cycle 10 of E:T ratio 20:1 and at cycle 20 of E:T ratio 10:1 (Fig 3A). *IL6* expression was significantly increased in all hypoxic and normoxic cells in comparison with non-co-cultured cells (PANC-1 controls) except normoxic cells after 20 cycles of hypoxia at an E:T ratio of 20:1. A statistically significant difference was noticed in PANC-1 cells exposed to 10 and 20 cycles of hypoxia relative to normoxic cells in an E:T ratio of 10:1 (Fig 3B). *TP53* expression was significantly increased in all co-cultured normoxic PANC-1 cells compared to non-co-cultured cells except at cycle 20 of E:T ratio 10:1. *TP53* expression was significantly decreased in all co-cultured hypoxic cells relative to hypoxic non-co-cultured cells in all conditions. *TP53* expression levels significantly increased in all normoxic co-cultured cells compared to hypoxic co-cultured cells (Fig 3C).

### Alterations in gene expression in hypoxic and normoxic PANC-1 cells treated with CD8+ T-cells CM

Instead of co-culture, we next measured gene expression of p53, TNF-α, and IL-6 in hypoxic and normoxic PANC-1 cells following treatment with either 100% or 75% CD8+ T-cells CM. PANC-1 cells treated with CD8+ T-cells CM showed a significant increase in *TNFα* gene expression, observed in all treated hypoxic and normoxic PANC-1 cells relative to untreated cells. A statistically significant difference was seen in TNF-α expression in hypoxic PANC-1 cells exposed to 10 cycles of hypoxia relative to normoxic cells in the 100% CM treatment. In contrast, no significant difference was observed in all other ratios (Fig 3D). *IL6* expression was significantly increased in all hypoxic and normoxic treated cells compared with untreated cells in a ratio-dependent manner except at cycle 20 and 75% CM ratio. Also, a statistically significant difference was noticed in hypoxic PANC-1 cells exposed to 10 cycles of hypoxia relative to normoxic cells in 100% and 75% CM ratios. However, when PANC-1 cells were exposed to 20 cycles of hypoxia, a significant difference in 100% CM ratio was observed relative to the normoxic cells (Fig 3E). On the other hand, *TP53* expression was significantly increased in all treated normoxic cells of cycle 10, with no significant difference in any of the treated normoxic cells at cycle 20. In hypoxic cells, *TP53* expression was significantly decreased in untreated hypoxic cells at all conditions. In comparison between treated hypoxic and normoxic cells, a significant decrease in *TP53* expression was observed in hypoxic cells treated with 100% CM ratio (Fig 3F).

### Cytokine production in CD8+ T-cells cultured in HCM or NCM of PANC-1 cells

**Fig 4:**
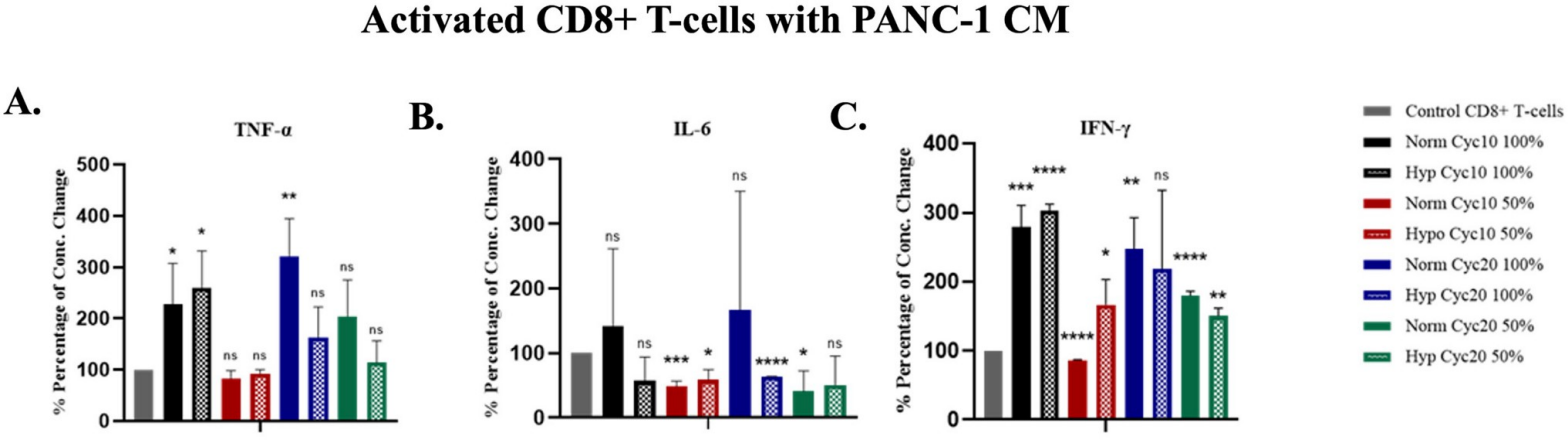
Percentages (%) of concentration changes of cytokines for CD8+ T-cells after cultured in HCM or NCM of PANC-1 cells. **A.** TNF-α P-value for treated CD8+ T-cells vs. control. **B.** IL-6 P-value for treated CD8+ T-cells vs. control. **C.** IFN-γ P-value for treated CD8+ T-cells vs. control. (*P<0.05, **P<0.01, ***P<0.001).

Next, we tested whether hypoxic PANC-1 cells can alter cytokine production from CD8+ T-cells. For that, we measured cytokine levels, TNF-α, IFN-γ and IL-6, after culturing activated T-cells in HCM or NCM for 72 hours in 100% and 50% ratios. TNF-α was significantly increased when cultured in 100% CM compared to control untreated cells, except for cells cultured in 100% HCM after PANC-1 cells were exposed to 20 cycles of hypoxia. On the other hand, TNF-α showed increased production when CD8+ T-cells cultured in HCM of PANC-1 cells exposed to 20 cycles of hypoxia at a 100% ratio in comparison with NCM with no significant difference between cells cultured on HCM or NCM in all other conditions (Fig 4A). For IL-6 production, significantly lower production was detected when cells were cultured in 50% NCM of cycle 10, 50% HCM of cycle 10, 50% NCM of cycle 20, and 100% HCM of cycle 20. However, no significant difference was observed in production between cells cultured in HCM or NCM in all culture conditions (Fig 4B). IFN-γ was significantly increased when cytotoxic cells were cultured in all ratios of HCM and NCM, except for cells cultured in 100% HCM after PANC-1 cells were exposed to 20 cycles of hypoxia. A significant reduction in IFN-γ production was measured when immune cells were cultured with 50% NCM of cycle 10 (Fig 4C). Also, a significantly higher production was detected when CD8+ T-cells cultured in HCM were exposed to 10 hypoxia cycles at a 50% ratio compared to NCM. However, lower production was detected when CD8+ T-cells cultured in HCM were exposed to 10 hypoxia at a 50% ratio in contrast with NCM (Fig 4C).

### Cytokines production in CD8+ T-cells and PANC-1 cells coculture

**Fig 5:**
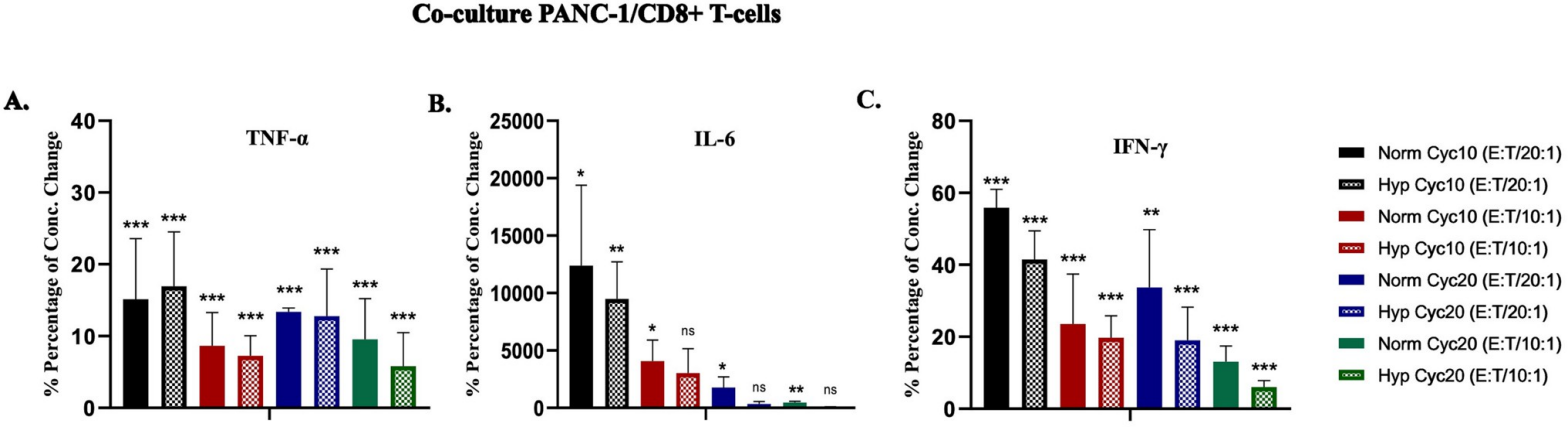
Percentages of concentration changes in TNF-α, IL-6, and IFN-γ in co-culture of hypoxic and normoxic PANC-1 cells with CD8+ T-cells. **A.** TNF-α P-value for treated PANC-1 cells vs. control. **B.** IL-6 P-value for treated PANC-1 cells vs. control. **C.** IFN-γ P-value for treated PANC-1 cells vs. control. (*P<0.05, **P<0.01, ***P<0.001).

TNF-α, IL-6, and IFN-γ production was measured using LEGENDplex Multi-Analyte Flow Assay Kit. The cytokine concentrations in untreated hypoxic and normoxic PANC-1 cells were lower than the detection limit. The control in this assay was the activated CD8+ T-cells in basic culture media RPMI-1640 (Fig 5). The concentration of TNF-α was lower than the control in a ratio-dependent manner. A statistically significant reduction in TNF-α concentration was observed in all co-culture conditions compared to the control. No significant difference was observed in TNF-α between hypoxic and normoxic PANC-1 cells after 10 and 20 cycles of hypoxia when co-cultured with CD8+ T-cells (Fig 5A). For IL-6, a statistically significant increase in its concentration was detected in all co-culture conditions compared to the control in a ratio-dependent manner. No significant difference was observed in IL-6 percentage between normoxic and hypoxic PANC-1 cells in all ratios except for hypoxic PANC-1 cells that underwent 20 cycles of hypoxia at an E:T ratio 10:1 (Fig 5B). Lastly, there was a statistically significant reduction in IFN-γ in all treated conditions compared to the control, but no difference was recorded between hypoxic and normoxic PANC-1 cells after 10 and 20 cycles of hypoxia (Fig 5C).

### Cytokines production in PANC-1 cells cultured in CD8+ T-cells conditioned media

**Fig 6:**
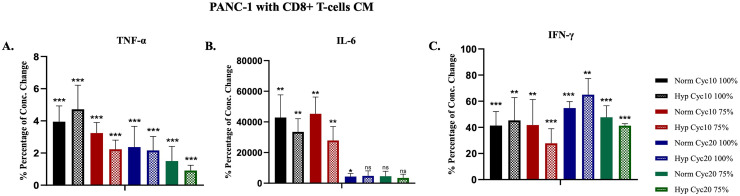
Percentages (%) of concentration changes in TNF-α, IL-6, and IFN-γ released by PANC-1 cells under hypoxic and normoxic conditions cultured with CD8+ T-cells CM. **A.** TNF-α P-value for treated PANC-1 cells vs. control. **B.** IL-6 P-value for treated PANC-1 cells vs. control. **C.** IFN-γ P-value for treated PANC-1 cells vs. control. (*P<0.05, **P<0.01, ***P<0.001).

CD8+ T-cells were activated for five days, and the CM was collected and cultured with hypoxic and normoxic PANC-1 cells for 72 hours. The supernatants were harvested, and the concentrations of the cytokines were measured. Then, the concentration changes in percentage relative to the control were evaluated in hypoxic and normoxic PANC-1 cells, and the results showed a lower level than the detection limit. The control in this assay was the CM cultured with PANC-1 cells. TNF-α concentration was not different between hypoxic and normoxic PANC-1 cells after 10 and 20 cycles of hypoxia were observed (Fig 6A). On the other hand, IL-6 concentration was higher in normoxic cells at 100% CM after 20 cycles. However, there was no significant difference in IL-6 percentage between normoxic and hypoxic PANC-1 cells exposed to 10 and 20 cycles of hypoxia at all ratios (Fig 6B). Lastly, there was no significant difference in IFN-γ production between hypoxic and normoxic PANC-1 cells after 10 and 20 cycles of hypoxia at all ratios (Fig 6C).

## Discussion

Despite the aggressive chemotherapeutic treatment, the overall survival rate of PC is still low. It is well-documented that hypoxia potentiates the development of chemoresistance in PC, inviting a better understanding of its contribution to TME [25,26]. Although immunotherapy has demonstrated promising outcomes in cancers such as melanoma and non-small cell lung cancer, it failed to generate similar effects in PC [26]. Subsequently, understanding the mechanisms leading to tumor cell evasion of immunotherapy is required. The present study focuses on studying pancreatic tumor cells under hypoxic conditions and their impact on cytotoxic CD8+ T-cell function, specifically, their cytokine-producing and tumor-cell-eliminating abilities. The hypoxia model used in this work was established previously, wherein hypoxia indicators in PANC-1 cells were validated at both the protein and gene expression levels [17–19].

Harnessing CD8+ T-cells in TME and reinforcing their cytotoxic activity against tumor cells is a cornerstone in cancer immunotherapy. In our study, culturing the activated CD8+ T-cells in 100% HCM or NCM of PANC-1 cells has increased cell proliferation compared to untreated control cells. Moreover, we observed increased TNF-α and IFN-γ production when cells were cultured in 100% of either HCM or NCM. In contrast, an overall reduction in IL-6 production was observed in CM-treated cells, with no significant difference between HCM and NCM. Contradictory to our results, previous studies have shown that cancer-derived factors have an immunosuppressive effect on different types of immune cells. For example, Abusamra *et al*. indicated that cancer exosomes suppressed T-cell proliferation and induced CD8+ T-cell apoptosis in a prostate cancer model [27]. In other studies, it was reported that cancer exosomes induce suppressor CD27- /CD28- CD8+ T-cells phenotype in head and neck cancer models [28] and inhibit the antigen-presenting function of dendritic cells in pancreatic cancer cell line model [29]. Interestingly, hypoxic and normoxic tumor secretomes were also shown to induce the suppressive effect on T-cells proliferation and IFN-γ production in a glioma stem cells model [30]. Notably, in this work, a clear identification of the source of these cytokines released in conditioned co-cultured media was not investigated, and further investigation is required to determine whether it is largely attributed to activated CD8+ T cells.

The suppressive effect of cancer secretomes on CD8+ T-cells can depend on the investigated tumor model. For example, Maybruck *et al*. showed that CD8+ T-cells were unaffected when cultured in colorectal cancer-conditioned media or exosomes [28]. In another study, when antigen-specific CTLs were co-cultured with a hypoxic NSCLC cell line, CTL reactivity was not affected, which could partly explain the results observed in this work [31]. Additionally, the ability of pancreatic tumor cells to escape immunosurveillance by T cells could be mediated through the expression of immune checkpoint ligands (e.g., PD-L1). However, this work did not investigate it [3]. A main finding in this work was inferred from co-culture experiments between activated CD8+ T-cells and hypoxic PANC-1 cells. Hypoxic cells exhibited a significantly higher resistance to the lytic effect of cytotoxic immune cells, as observed by the MTT assay. The hypoxic PANC-1 cells showed a significant resistance at (E:T ratio of 20:1 and 10:1) after ten cycles of hypoxia (P-values = 0.002, 0.004 respectively) and at all ratios after 20 cycles of hypoxia (P-values = 0.002, 0.007, 0.019 for E:T ratio 20:1, 10:1, and 5:1 respectively). A significant reduction in TNF-α and IFN-γ secreted levels accompanied this effect. Many explanations may be correlated to these results. First, the inhibition of CD8+ T-cells in direct contact with PANC-1 cells may occur, reducing its ability to produce cytokines. Second, cancer cells may produce proteases that shed the membrane-bound receptors to produce soluble receptors, which act as inhibitors for their soluble proteins [32]. The metalloproteinase ADAM8 is highly expressed in PDAC and causes TNFR-1 shedding [33,34]. Third, cancer cells may also produce factors that affect TNF-α and IFN-γ stability as a mechanism of resistance, such as the metalloproteinase ADAM17, which degrades IFN-γ in MCF-7 and MDA-MB-453 breast cancer cell lines [35].

Notably, TNF-α gene expression was increased in all treated PANC-1 cells. TNF-α expression may be increased due to the ability of its membrane-bound form to act as a receptor by transmitting a reverse signal back to its bearing cells when bound to TNFR [36]. The binding of TNF-α to TNFR2 was shown to contribute to the immunosuppressive effects in the TME and promoted cancer cell survival via the NF-κB pathway [37]. Lastly, IL-6 expression was significantly increased in all treated PANC-1 cells. A statistically significant difference was noticed between hypoxic and normoxic PANC-1 cells, suggesting that IL-6 expression increased in stress conditions as a resistance mechanism in cancer cells. Importantly, data in this work was limited to a single tumour cell line; thus, these observations can still be cell-line dependent. Future work should focus on validating them in other pancreatic tumor cell lines.

p53 is a tumor suppressor transcription factor that regulates cellular apoptosis and metabolism. *TP53* expression was increased in PANC-1 cells after exposure to hypoxic shots. In addition, *TP53* expression was significantly increased in all treated normoxic PANC-1 cells and decreased in all treated hypoxic cells. *TP53* is mutated in approximately 50% of all cancer types. The regulation of immune cell interaction and response to hypoxic conditions has been suggested to be a function of the mutated p53 [38]. Subsequently, the increase in p53 expression in PANC-1 cells might serve as an additional resistance mechanism after treating PANC1 cells with different concentrations of HCM. In the study of Hashimoto et al., it was found that mutated p53 protein was associated with the PD-L1 recycling process and cell surface expression in pancreatic cancer [39]. Furthermore, it was reported that some versions of the mutated p53 promoted NF-κB transcriptional function, which upregulates the transcription of TNF-α and IL-6 [40]. Lastly, it is important to note that our analysis involved measuring the gene expression level of these three molecular players, which could have overlooked certain posttranslational modifications.

In the TME, some tumor cells are not in direct contact with the cytotoxic immune cells and their interaction is mediated in a non-cell-autonomous fashion. For that, we cultured the PANC-1 cells with CD8+ T-cells CM and ran the same assays as in the co-culture experiments. MTT results showed that the hypoxic PANC-1 cells display statistically significant resistance to CD8+ T-cells secretory proteins among 100%, 75%, and 50% ratios of CM after being exposed to 10 cycles of hypoxia and among all ratios of exposure to 20 cycles of hypoxia. Moreover, TNF-α and IFN-γ concentrations were decreased in a ratio-dependent manner in all treated PANC-1 cells, with no significant difference between the hypoxic and normoxic cells. These results support our suggestion that PANC-1 cells may produce factors or proteases that affect TNF-α and IFN-γ stability as a resistance mechanism. This mechanism may have occurred with or without an inhibitory effect on CD8+ T-cells in direct co-culture. For IL-6 production, a significant sharp increase in IL-6 concentrations was observed in all treated PANC-1 cells, and no significant differences were observed between treated hypoxic and normoxic cells.

## Conclusion

In conclusion, our cell viability results demonstrate that hypoxic PANC-1 cells exhibit increased resistance to the cytotoxic effects of CD8+ T-cells. This resistance is accompanied by a significant reduction in the levels of secreted TNF-α and IFN-γ. This suggests that the inhibition of CD8+ T-cells in direct contact with PANC-1 cells may be due to a decreased ability to produce cytokines, or possibly because cancer cells produce proteases that shed membrane-bound receptors, leading to the formation of soluble receptors that act as inhibitors. Additionally, cancer cells may produce factors that destabilize TNF-α and IFN-γ, contributing to resistance. A significant difference in IL-6 expression was also observed between hypoxic and normoxic PANC-1 cells when cultured with CD8+ T-cell conditioned media, indicating that this cytokine could be a key factor in the altered resistance mechanisms of hypoxic cells. Further research is needed to explore strategies for overcoming hypoxia-induced resistance and to validate these findings across different pancreatic tumor cell lines, which could provide valuable insights for improving clinical outcomes.

## Supporting information

S1 Table Primer sequences. (DOCX)

S1 Fig CD8+ T-cells percentage in PBMCs and purity after isolation and activation. **A)** CD8+ T-cells percentage in the PBMCs and eluted unwanted cells after isolation **B)** Stained CD8+ T-cells with CD8/CD3/CD4 antibodies after isolation. **C)** CD8+ T-cells purity after three days of activation and staining with CD8 and CD4 antibodies. **D)** CD8+ T-cells purity after five days of activation.
(TIFF)

S2 Fig Assessment of CD8+ T-cells viability after activation and culturing in HCM vs. NCM for 72 hours by staining with 7-AAD. **A.** Unstained CD8+ T-cells with 7-AAD. **B.** Stained control cells cultured in RPMI-1640 media. **C.** CD8+ T-cells cultured in 50% HCM of cycle 10. **D.** CD8+ T-cells cultured in 100% HCM of cycle 10. **E.** CD8+ T-cells cultured in 50% HCM of cycle 20. **F.** CD8+ T-cells cultured in 100% HCM of cycle 20. **G.** CD8+ T-cells cultured in 50% NCM of cycle 10. **H.** CD8+ T-cells cultured in 100% NCM of cycle 10. **I.** CD8+ T-cells are cultured in 50% NCM of cycle 20. **J.** CD8+ T-cells cultured in 100% NCM of cycle 20.
(TIFF)
