# Supplementary material for: Effect of cytotoxic CD8+ T-cells secretory proteins on hypoxic pancreatic cancer cells
Source: PLoS One. 2025 Jan 30;20(1):e0311615. doi: 10.1371/journal.pone.0311615 (PMC11781647; doi:10.1371/journal.pone.0311615)
Supplement: S1 Table — (DOCX) [file pone.0311615.s001.docx]

| Gene | Forward 5΄-3΄ | Reverse 5΄-3΄ | Annealing Temperature |
| --- | --- | --- | --- |
| P53 | GGCTCTGACTGTACCACCAT | CACCTCAAAGCTGTTCCG TC | 65.3 ͦC |
| TNF-α | GTCAACCTCCTCTCTGCCAT | CCAAAGTAGACCTGCCCAGA | 61.0 ͦC |
| BAX | CCTTTTCTACTTTGCCAGCAAAC | GAGCCCGTCCCAACCAC | 60.0 ͦC |
| IL-6 | TTCCAAAGATGTAGCCGCCC | ACCAGGCAAGTCTCCTCATT | 57.7 ͦC |
| GAPDH | CCTGTTCGACAGTCAGCCG | CGACCAAATCCGTTGACTCC | 60 ͦC |
