# Supplementary figures and images for: Effect of cytotoxic CD8+ T-cells secretory proteins on hypoxic pancreatic cancer cells
Source: PLoS One. 2025 Jan 30;20(1):e0311615. doi: 10.1371/journal.pone.0311615 (PMC11781647; doi:10.1371/journal.pone.0311615)

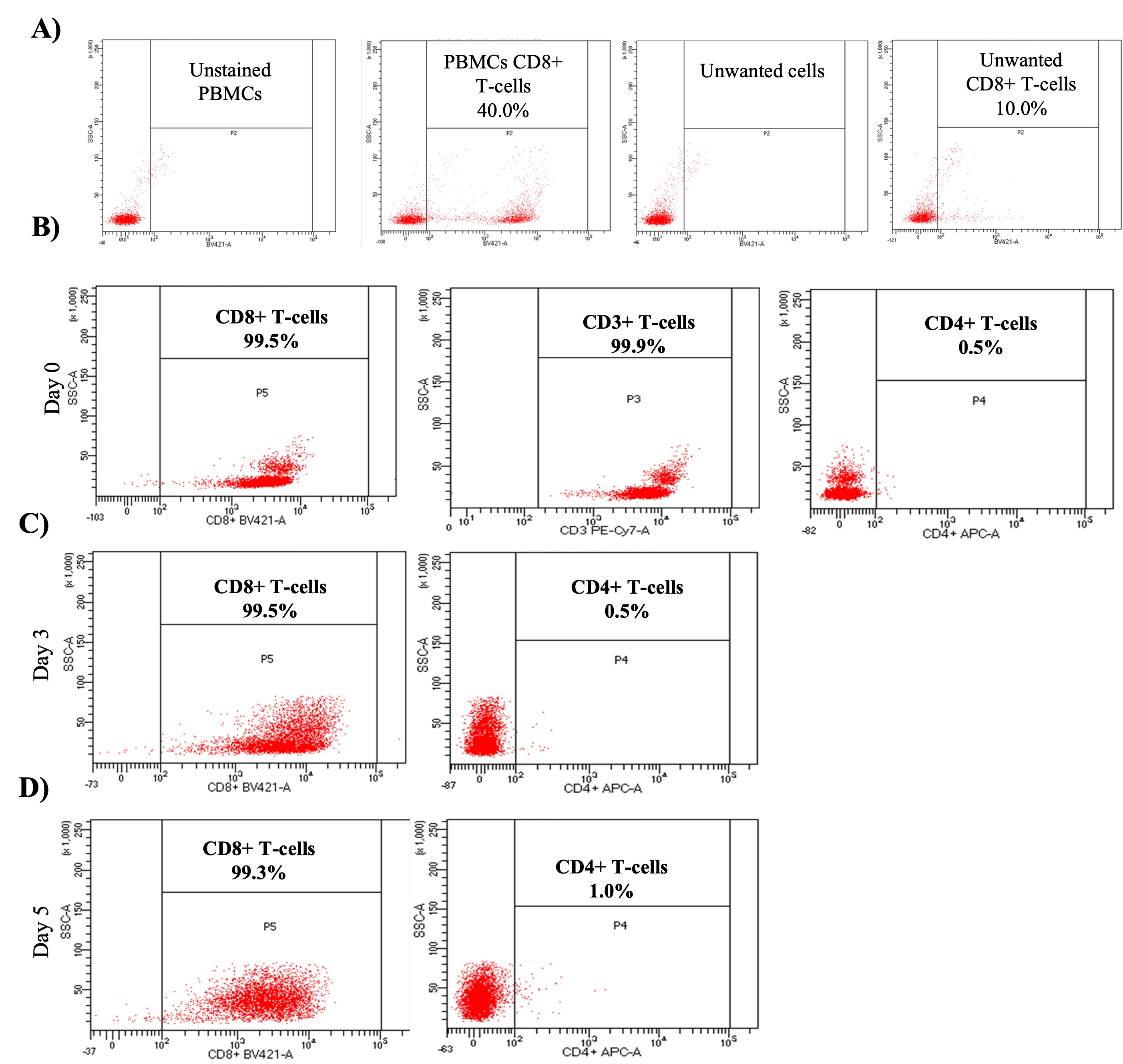

Supplement: S1 Fig — A) CD8+ T-cells percentage in the PBMCs and eluted unwanted cells after isolation B) Stained CD8+ T-cells with CD8/CD3/CD4 antibodies after isolation. C) CD8+ T-cells purity after three days of activation and staining with CD8 and CD4 antibodies. D) CD8+ T-cells purity after five days of activation. (TIFF) [file pone.0311615.s002.tiff]

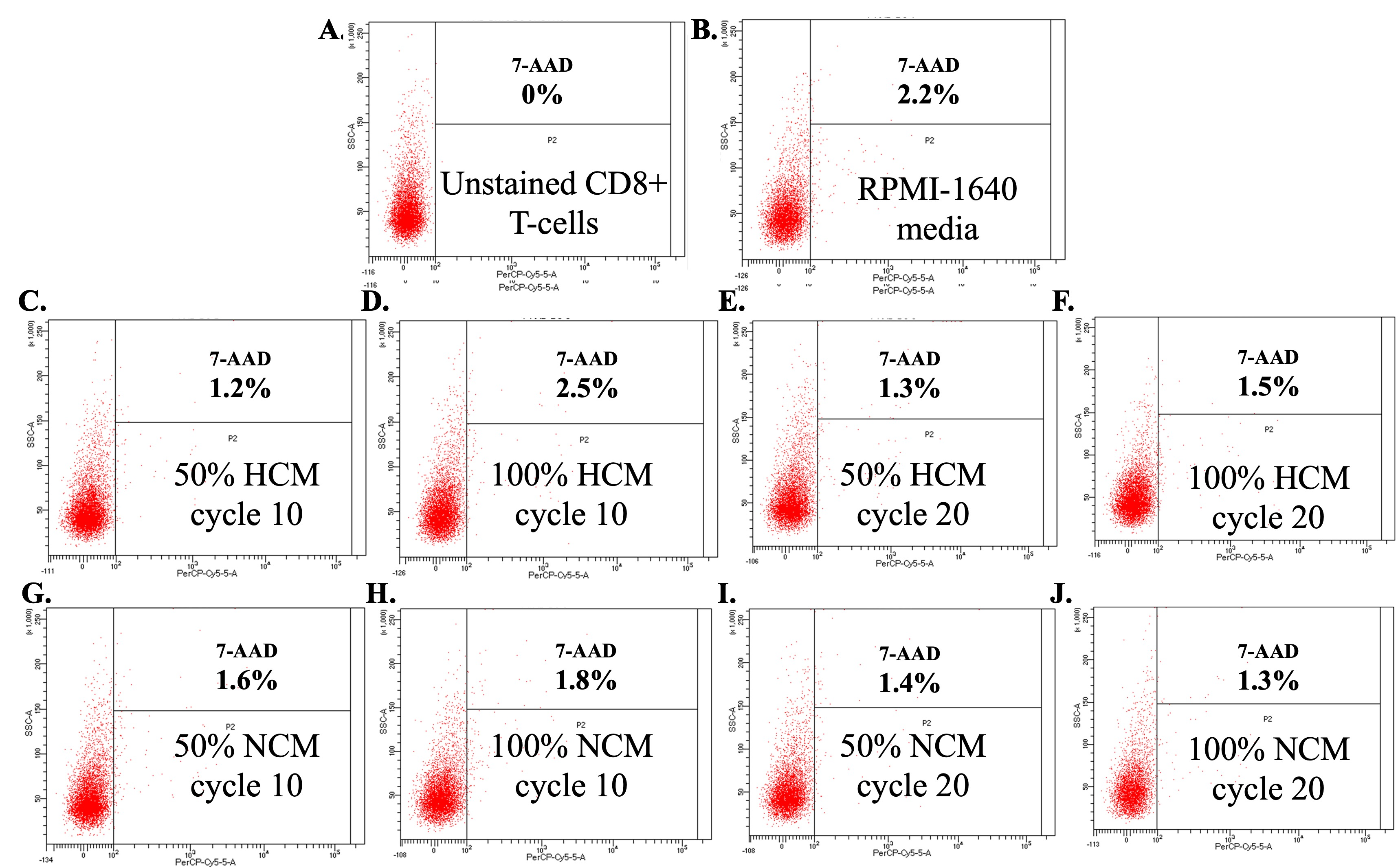

Supplement: S2 Fig — A. Unstained CD8+ T-cells with 7-AAD. B. Stained control cells cultured in RPMI-1640 media. C. CD8+ T-cells cultured in 50% HCM of cycle 10. D. CD8+ T-cells cultured in 100% HCM of cycle 10. E. CD8+ T-cells cultured in 50% HCM of cycle 20. F. CD8+ T-cells cultured in 100% HCM of cycle 20. G. CD8+ T-cells cultured in 50% NCM of cycle 10. H. CD8+ T-cells cultured in 100% NCM of cycle 10. I. CD8+ T-cells are cultured in 50% NCM of cycle 20. J. CD8+ T-cells cultured in 100% NCM of cycle 20. (TIFF) [file pone.0311615.s003.tiff]
